# Pregnancy in Primary Biliary Cholangitis: A Single-Center Experience Emphasizing the Need for Postpartum Monitoring

**DOI:** 10.5152/tjg.2026.26054

**Published:** 2026-07-06

**Authors:** Ersin Batıbay, Mahmut Polat, Akın Kaya, Ahmet Tay

**Affiliations:** Department of Gastroenterology, Harran University Faculty of Medicine, Şanlıurfa, Türkiye

**Dear Editor,**

We read with great interest the invited review by Ergenç et al,^1^ “Preexisting Liver Disease and Pregnancy: Optimizing Care to Optimize Outcomes,” recently published in the *Turkish Journal of Gastroenterology*. The authors highlight the importance of preconception counseling, trimester-based monitoring, and multidisciplinary management to optimize maternal and fetal outcomes in women with chronic liver diseases.^1^ Notably, they emphasize that disease-specific vulnerabilities may emerge during pregnancy and particularly in the postpartum period.^1^

Primary biliary cholangitis (PBC) is a chronic autoimmune cholestatic liver disease that predominantly affects women. Although most patients are diagnosed later in life, clinically relevant proportions of patients are identified during their reproductive years, necessitating evidence-based counseling regarding pregnancy safety, fetal outcomes, and postpartum disease activity.^2^^,^^3^

Pregnancy is generally well tolerated in PBC; however, rates of miscarriage and preterm delivery may be higher than in the general population, and postpartum biochemical flares are relatively common.^4^^,^^5^ Previous studies have reported postpartum flare rates up to 60%, underscoring the need for structured postnatal follow-up.^6^

Biochemical flares were defined as a ≥2-fold increase in aminotransferases and/or a ≥3-fold increase in alkaline phosphatase compared with baseline values.^6^ Furthermore, previous studies suggest that biochemical changes during pregnancy may occur particularly during the second and third trimesters, reflecting dynamic immunological and hormonal adaptations throughout gestation.^4^^-^^6^

To provide additional data aligned with the clinical priorities highlighted by Ergenç et al,^1^ we retrospectively evaluated 6 women with PBC who experienced 12 pregnancies at Harran University Faculty of Medicine.

The mean maternal age at pregnancy was 34.4 years (range 25-41 years), and the mean duration of PBC prior to pregnancy was 4.6 years (range 0-9 years).

Baseline disease severity was generally mild. AMA-M2 positivity had been documented before pregnancy in 5 patients, whereas 1 patient was diagnosed during pregnancy following evaluation for pruritus and positive AMA-M2 serology. The median preconception alkaline phosphatase level was 153 U/L (range 72-232 U/L). Preconception FibroScan assessment demonstrated F0-F1 fibrosis in 4 patients and F2 fibrosis in 2 patients. Baseline liver biopsy data were available in 2 patients, whereas such information was unavailable for the remaining patients because several had been diagnosed and followed at external centers. Importantly, none of the patients had clinical, laboratory, or radiological evidence of cirrhosis before pregnancy or during postpartum follow-up.

Most patients (83.3%, n = 5) had an established diagnosis prior to conception, whereas 1 patient (16.7%, n = 1) was diagnosed during pregnancy.

Biochemical flares occurred in 25% (n = 3) of pregnancies, whereas postpartum flares were observed in 58.3% (n = 7), supporting the postpartum period as the most vulnerable phase for disease activity.^6^

In this cohort, postpartum flares occurred within the first 3-6 months after delivery. New-onset or worsening pruritus occurred in 41.7% (n = 5) of pregnancies.

Regarding fetal outcomes, miscarriage occurred in 25% (n = 3) and preterm delivery occurred in 8.3% (n = 1). There were no stillbirths and no fetal anomalies. No maternal deaths occurred, and no patients required liver transplantation during the postpartum period.

Ursodeoxycholic acid (UDCA) therapy was continued throughout pregnancy in 8 of 12 pregnancies (66.6%) and during lactation in 9 pregnancies (75%), without documented adverse events. UDCA was administered at standard weight-based doses (13-15 mg/kg/day). Treatment was discontinued in 4 pregnancies; 3 discontinuations were based on patient preference and 1 followed recommendations received during obstetric consultation.

Current guidance supports UDCA as standard therapy for PBC management.^7^

All patients had isolated PBC without features suggestive of autoimmune hepatitis overlap, and no patients required corticosteroid therapy during pregnancy or postpartum flares. In cases of persistent pruritus, additional symptomatic treatments such as rifampicin or cholestyramine were occasionally used.

Among the 7 pregnancies complicated by postpartum flare, 5 occurred despite ongoing UDCA therapy, whereas 2 developed after treatment discontinuation. Two of these pregnancies also had a history of flare during a previous pregnancy. In all pregnancies complicated by postpartum flare, UDCA therapy was continued or reintroduced when previously discontinued, according to clinical assessment.

With the exception of 1 patient, biochemical flares were controlled within 6 months postpartum.

Patients were monitored with periodic liver biochemical tests throughout pregnancy. Laboratory assessments were performed approximately every 3 months during the first 2 trimesters and monthly during the third trimester and during the first 3 months postpartum.

The findings of the current study reinforce the need to integrate structured postpartum monitoring into pregnancy care pathways for women with PBC.

These observations are consistent with previous reports indicating that disease activity in autoimmune liver diseases may fluctuate during pregnancy and the postpartum period. Hormonal and immunological changes during gestation may partially modulate autoimmune activity. In contrast, the postpartum period is often associated with immune reconstitution and an increased risk of biochemical flare.^4^^-^^6^

In this context, recent literature emphasizes the importance of multidisciplinary management and structured monitoring strategies in women with preexisting liver disease throughout pregnancy and the postpartum period, as highlighted by Ergenç et al.^1^

The small sample size is an important limitation, and the findings should be considered hypothesis generating.

In conclusion, pregnancy in well-controlled PBC appears largely feasible, but the postpartum period remains a predictable high-risk phase for biochemical flare.^6^ Larger multicenter studies are needed to refine monitoring strategies and define optimal postpartum follow-up intervals.

## Data Availability

The data that support the findings of this study are available on request from the corresponding author.
